# Supplementary material for: Study on the Technology of Monodisperse Droplets by a High-Throughput and Instant-Mixing Droplet Microfluidic System
Source: Materials (Basel). 2021 Mar 7;14(5):1263. doi: 10.3390/ma14051263 (PMC7962067; doi:10.3390/ma14051263)
Supplement: Supplementary file 1 [file materials-14-01263-s001.pdf]

materials

Supplementary Mterials

Study on the Technology of Monodisperse Droplets by a High-Throughput and Instant-Mixing Droplet Microfluidic System

Rui Xu, Shijiao Zhao, Lei Nie, Changsheng Deng, Shaochang Hao, Xingyu Zhao, Jianjun Li, Bing Liu and Jingtao Ma

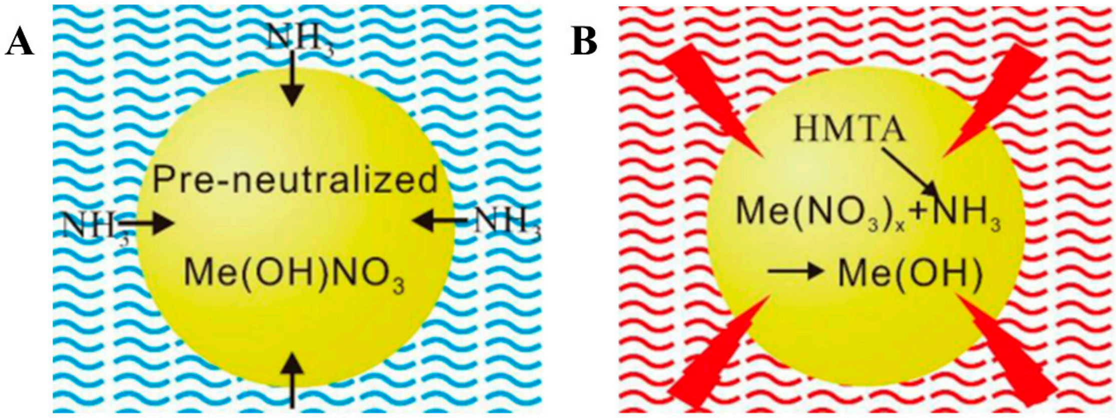

Figure S1. (**A**) The external gelation process; (**B**) The internal gelation process.

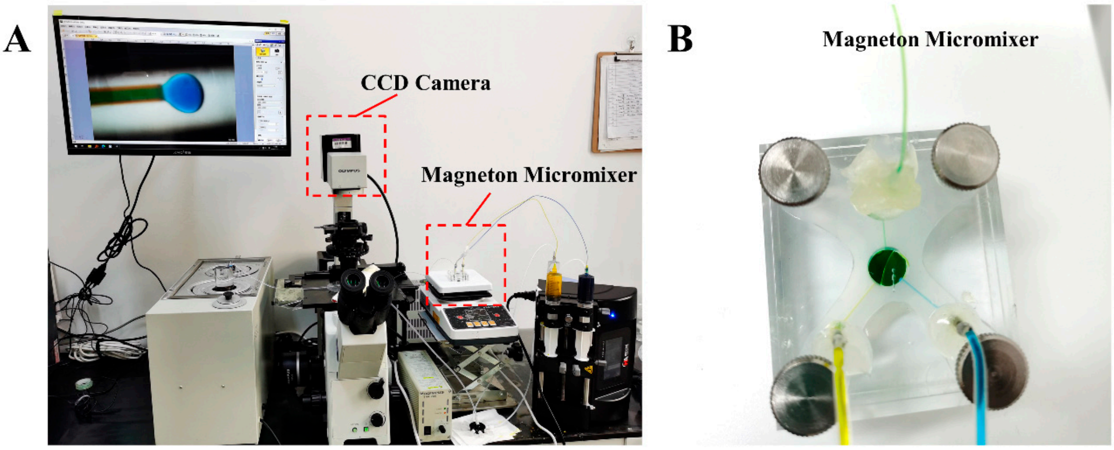

Figure S2. (**A**) Physical image of the high-throughput and instant mixing droplet microfluidic system; (**B**) The physical image of the magneton micromixer.
